# Mechanical Property and Microcellular Foamability of iPP/PA11/PP-g-MAH Blends

**DOI:** 10.3390/polym17141952

**Published:** 2025-07-16

**Authors:** Bosi Liu, Yangzheng Wang, Jingke Pei, Qiongdan Fan, Kun Li, Lele Li, Xiaoli Zhang

**Affiliations:** 1 School of Materials Science and Engineering, National Engineering Research Center for Advanced Polymer Processing Technology, Zhengzhou University, Zhengzhou 450001, China; bosi4162022@163.com (B.L.); wangyangzheng622@163.com (Y.W.); fanqiongdan0916@163.com (Q.F.); kunli@zzu.edu.cn (K.L.); 2 Sinopec (Henan) Refining and Chemical Co., Ltd., Luoyang 471012, China; 3 Dongfang Electric (Fujian) Innovation Institute Co., Ltd., Fuzhou 350108, China; peijingke12321@163.com

**Keywords:** isotactic polypropylene, compatibility, mechanical property, microcellular foaming

## Abstract

To improve the mechanical property and foamability of linear structured isotactic polypropylene (iPP), a second phase of polyamide11 (PA11) was introduced to the iPP matrix, and a low contented PP-g-MAH was added to adjust their compatibility. As a result, a high impact strength of 8.43 kJ/m^2^ (a 118% increase compared to that of iPP) and an elongation at break of 465.87% (a 130% increase compared to that of iPP) of the compounded iPP/20PA11/10PP-g-MAH were achieved, which was attributed to the PA11 being well distributed in the iPP matrix and to the compatibility enhancement by PP-g-MAH. Depending on a suitable material formulation and a bath foaming strategic design, microcellular cells with an average size from 204.8 to 5.9 μm and a cell density from 6.0 × 10^6^ to 6.5 × 10^9^ cells/cm^3^ were obtained. Due to the significant enhancement of melt strength by partially melted crystals, combined with the synergistic effect of PA11, a quiet high expansion ratio of up to 37.9 was achieved. These manufactured foams have potential applications in packaging, thermal insulation, and other industrial fields.

## 1. Introduction

Isotactic polypropylene (iPP) is one of the most widely used universal semi-crystalline polymers in industry, due to its relatively low price, good chemical resistance, and thermal stability, as well as its nontoxic feature. On account of its aforementioned advantages, iPP has been widely applied in packaging, medical instruments, household and electrical appliance parts, civil architecture, and so on [1,2,3,4]. Recently, microcellular semi-crystalline polymers have attracted growing attention, as they can reduce the cost and density of the products while maintaining their desired performances, such as excellent heat resistance and fine impact strength. Polymeric iPP-based microcellular foams have good comprehensive mechanical properties and are potentially applied in some areas, compared to their traditional foamed counterparts [5,6,7,8]. Supercritical Fluids of carbon dioxide (CO_2_) or nitrogen (N_2_) are usually used as physical foaming agents during polymer’s physical foaming process [9,10,11,12]. Microcellular foamed polymers are now popularly used in the areas of packaging, automobile industry, sports, shoes, daily applicants, and so on [13,14,15,16]. However, the foamability of iPP is quite poor due to its fast crystallization characteristics and low melt strength, under a melting state processing condition, which predominantly hinder the development and application of microcellular foamed iPPs [17,18,19].

IPP is a linear structured polymer material with a few branched molecular chains and a relatively narrow molecular weight distribution, whose softening and melting points are comparatively close to each other. When it is heated during foaming, iPP basically does not flow before reaching its melting point (T_m_), while reaching the T_m_, the melt strength of iPP drops sharply and makes it difficult to encapsulate the foaming gases. Moreover, when iPP transitions from the molten state to the crystallized state, a large amount of heat will be released, which takes it a long time to be transformed into a solid state. In addition, iPP has a high permeability, so the foaming gas is prone to escape from the melt. Therefore, the temperature range suitable for iPP foaming is rather narrow, and the foaming process is difficult to control, which may easily lead to bubble wall rupture, collapse, or merging in the final foaming stage [20]. Furthermore, due to the physical foaming agent, usually the CO_2_ or N_2_ gas, is not able to be dissolved in the crystalline region of the iPP, the foaming process will be inherently non-uniform [7]. Thus, a reasonable control of crystallization behaviors and improving the melt strength of iPP are the keys to obtain high-quality iPP foams.

Theoretically, increasing molecular weight and its distribution, introducing long branching structures, and grafting or adding inorganic nanoparticles can usually achieve the purpose of enhancing the melt strength of a semi-crystalline polymer [19,21,22]. Nevertheless, the above methods need to develop specially customized iPP models or introduce other processes, which undoubtedly increases the production cost and is not conducive to industrial promotion. Blending iPP with another polymer phase is currently one of the most efficient and feasible methods for enhancing its melt strength and foamability [11,23,24,25,26,27,28,29].

McCallum et al. [18] studied the effects of branched PP on the improvement of crystallinity and mechanical properties of iPP. Rizvi et al. [30] explored the role of in situ manufactured polyethylene terephthalate (PET) fibrils in the rheological and crystallization properties of linear polypropylene (PP). Dehghan et al. [31] reported the influence of hybrid nanofillers of MXene and reduced graphene oxide on the enhancement of iPP’s foaming ability; as a result, the size of the cells became smaller, leading to a slight increase in cell densities, i.e., the cell size (8.01–13.3 μm) of PP/MXene: rGO nano composite was smaller than that of PP/MXene ones (9.7–15.2 μm). Yang et al. [32] applied a γ-ray radiation crosslinking and a second phase of high-density polyethylene (HDPE) to enhance the foaming behavior of iPP; a broadened foaming temperature window of 8–12 °C and smaller-sized cells with high cell density resulted. In our previous studies, based on the understanding of partially melted crystals of semi-crystallized polymers, the self-enhancement effect of those partially melted structures on iPP’s foamability was confirmed to be effective [7,20,33,34,35].

Polyamide 11 (PA11), which is a semi-crystalline polymer with both ordered and disordered phases, has the advantages of low density, high impact strength, and good dimensional stability. In order to enhance the toughness of iPP and improve its foaming ability, PA11 can be added to iPP for the purpose of blending modification [36,37]. Fu et al. [38] reported that the addition of PA6 positively affected the crystallization, mechanical, and foaming properties of PP; additionally, a pressure-induced flow processing could further dramatically enhance such effects. However, the significant polarity difference between PA11 and iPP can generally result in their extremely poor compatibility. At the same time, the appropriate blending ratio and process parameters are also important factors to affect the foaming performance of this blend. Therefore, it is necessary to conduct extensive experiments to find the ideal solution for microcellular foaming of PP/PA11 composites.

In this study, a second phase of PA11 was introduced into the iPP matrix to modify its melt strength, and a low-content PP-g-MAH (a grafted PP) was used to improve the compatibility of iPP and PA11. Moreover, partially melted iPP crystal structures were designed through setting suitable foaming temperature to enhance the foaming behavior of the iPP/PA11/PP-g-MAH blends. The compatibility, thermal properties, crystallization and rheological behaviors, cellular structure, and mechanical properties of the composites were studied in detail. Cooperating with the reinforcement of the melt strength and cell nucleating sites of PA11, in our previous study, partially melted crystals in semi-crystalline polymers not only increase its melt strength, but also present a role in generating the cell nucleating effect, and thus produce ideal microcellular foams. This study provides an easy strategy for fabricating high-quality iPP/PA11/PP-g-MAH microcellular foams.

## 2. Materials and Methods

### 2.1. Materials

The linear iPP (Trade Number of T30S) used in this study was manufactured by Ningxia Petrochemical Company (Ningxia, China). The density of the iPP is 0.91 g/cm^3^ and the melt flow index (MFI) is 2.1 g/10 min (230 °C, 2.16 kg). The nominal melting temperature of this iPP is 167 °C.

The PA11 (Trade Number of BMNO TLD) was produced by the Atochem Company (Lyon, France) and has a density of 1.03 g/cm^3^, a melt flow index of 36.5 g/10 min (235 °C, 2.16 kg), and a melting temperature of 189 °C. The water absorption rate of this PA11 is 1.6%, so it should be thoroughly dried before processing.

PP-g-MAH (Trade number of 50E806) was produced by the Dupond Company (Wilmington, DE, USA) and has a density of 0.9 g/cm^3^, a melt flow index of 100.0 g/10 min (190 °C, 2.16 kg), and a melting temperature of 165 °C.

A physical foaming agent of carbon dioxide (CO_2_) with a purity of 99.5% was purchased from Qiangyuan Chemical Industry Company (Zhengzhou, China).

### 2.2. Sample Preparation

The IPP pellets were dried in a vacuum oven at 70 °C for 6 h, and the PA11s were dried at the same temperature for at least 10 h. The weight ratio of iPP/PA11 is respectively 100:0, 95:5, 90:10, 85:15, and 80:20 without PP-g-MAH; the another group is for a given weight of 10% PP-g-MAH; and the iPP/PA11 is 85:5, 80:10, 75:15, and 70:20, respectively. For example, an iPP/20PA11/10 PP-g-MAH means that 20% PA11 and 10% PP-g-MAH weighted were added into the iPP matrix; the others are marked similarly. The dried and weighted materials were compounded together using a mini opposite rotated twin screw extruder at a rotating speed of 80 rpm and a processing temperature of 195 °C. The extrudates were cut with a scissors and then pressed by a laboratory typed press from Xinben Company (Qingdao, China), at a pressure of 10 MPa and a temperature of 195 °C. During the pressing, a rectangle mold was used and the produced compounds were further applied for different characteristics and batch foaming.

### 2.3. Batch Foaming Experiment

**Figure 1:**
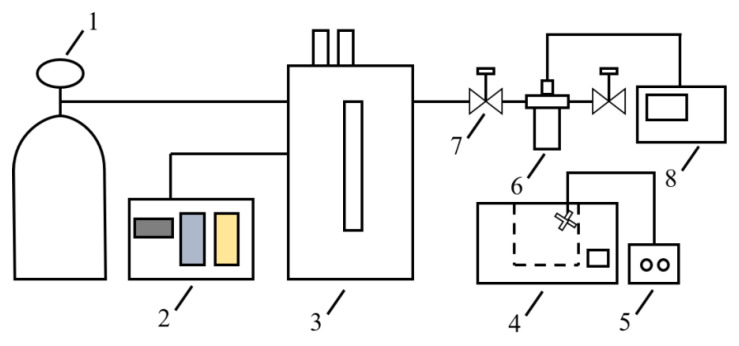
Scheme of batch foaming equipment. 1—CO_2_ gas tank, 2—Pump controller, 3—Syringe pump, 4—Oil heater, 5—Electric mixer, 6—Pressurized autoclave, 7—Vale, 8—Temperature control device.

As shown in Figure 1, iPP/PA11 and iPP//PA11/PP-g-MAH samples were foamed in a high pressurized autoclave with a volume of 50 mL. The temperature was controlled by an oil bath with an accuracy of ±0.5 °C. The foaming pressure was adjusted by a syringe pump (2ZB-2L20A), purchased from the Beijing Satellite Instrument Company (Beijing, China).

According to our previous studies about microcellular foaming of iPP and its composites, a foaming strategy with a fixed pressure of 12 MPa and a saturation time of 30 min, but different temperatures of 156 °C, 158 °C, 160 °C, 162 °C, and 164 °C, was designed and applied [7,20,33].

### 2.4. Differential Scanning Calorimetry (DSC) Analysis

A Differential Scanning Calorimeter (DSC3, Mettler Toledo, Zurich, Switzerland) was applied to measure the thermal property of iPP and its composites. Samples with a weight of about 8 mg were heated from 20 °C to 200 °C, at a heating rate of 10 °C/min. After holding for 2 min, the samples were then cooled to 20 °C, using a cooling rate of 10 °C/min, protected by the nitrogen gas. Based on the tested results, the crystallinity of iPP or PA11 ($\chi_{c}$) was calculated using the following Equation (1).

(1) $$\chi_{c}=\frac{{∆H}_{m}}{{∆H}_{m}^{0}}\times 100\%$$

where ${∆H}_{m}$ is the melting enthalpy of iPP or PA11, and ${∆H}_{m}^{0}$ is the 100% crystallization enthalpy of iPP or PA11, which is 209.0 J/g or 195.0 J/g, respectively [38,39,40].

### 2.5. X-Ray Diffraction Test

A SmartLab SE X-ray diffraction tester (Rigaku, Shojima City, Tokyo, Japan) was used to measure the crystal structure of the composites within a scope of 5–30°; the sweeping speed was 5°/min, and the sample thickness was 1.5 mm.

### 2.6. Fourier Transform Infrared Spectroscopy (FTIR)

Infrared spectrums of iPP/PA11/PP-g-MAH blends were measured by an infrared spectrometer IRTracer 100 (Shimadzu Corporation, Kyoto, Japan), using a reflection ATR model; the tested scope was 4000–400 cm^−1^, and the disc-shaped sample had a diameter of 24 mm, with a thickness of 1.5 mm.

### 2.7. Rheological Characterization

To study the effects of POMS and PA11 on the iPP’s rheological properties, a rotational rheometer of the Bohlin Gemini model (Malvern Corporation, Milton Keynes, UK) was used for rheological tests. The disc-shaped samples with a diameter of 24.0 mm and a thickness of 1.0 mm were compressed using the same press mentioned in Section 2.2; the distance between the two plates was originally set to 1.1 mm, and after the loading of the sample, the upper plate was lowered. The sample was heated from room temperature to 200 °C and held for at least 3 min; thus, the influence of iPP crystallized structures was successfully eliminated, so we could observe the effect of PA11 addition. When the scanning operation was started, a given strain of 1.0%, frequency from 0.1 to 100 rad/s were used.

### 2.8. Scanning Electron Microscopy (SEM)

A Scanning Electron Microscope (Quanta 200, FEI Company, Hillsboro, OR, USA) was employed to measure the morphology of the fractured composite and cell morphology of the iPP based foams. The samples were fractured after being immersed in liquid nitrogen for at least 60 min to ensure the success broken of the composites. The fractured surface was covered with the Au (Aurum) films before the SEM test. Moreover, based on the obtained SEM pictures, the cell parameters were analyzed using the Image J software 1.54p.

The expansion ratio ($\varphi_{v}$) can be calculated using the following Formula (2).

(2) $$\varphi_{v}=\frac{\rho_{0}}{\rho_{f}}$$

where $\rho_{0}$ and $\rho_{f}$ are the densities of un-foamed and foamed iPP, respectively. Both of them were obtained using the water displacement method.

The cell size can be evaluated using the software of Image J, and the cell density (*N*) can be calculated by the following Equation (3).

(3) N=(nA)32×φv

where *n* is the cell number in the SEM image selected, and *A* is the chosen area of the image.

### 2.9. Mechanical Test

Non-foamed iPP and iPP/PA11//PP-g-MAH specimens with a length of 80 ± 2 mm, a width of 10 ± 0.2 mm, and a thickness of 4 ± 0.2 mm were produced by an injection molding machine (WZS10D, Xinshuo Precision Machinery Co., Ltd., Shanghai, China). Moreover, a 1.5 mm V-shaped depth was cut in these samples, and an impact tester (PTM7000, Sansi Company, Shenzhen City, China) was applied for an impact test. The tensile test was conducted using the universal tensile tester (UTM6104, Sansi Company, Shenzhen City, China) with a speed of 50 mm/min. Five samples were prepared and applied for each mechanical measurement.

## 3. Results and Discussion

### 3.1. Thermal and Rheological Properties of iPP/PA11/PP-g-MAH

**Figure 2:**
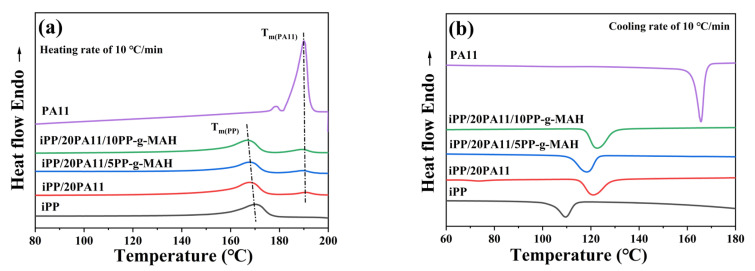
DSC melting curves (**a**) and cooling curves (**b**) of iPP/20PA11/PP-g-MAH blends with different PP-g-MAH content.

The melting and cooling points of iPP and its composites are critical information for setting their processing parameters. Figure 2 presents the melting and cooling curves of iPP and its PA11 blends. As illustrated in Figure 2a and Table 1, the melting point of the iPP is 169.2 °C, but that of the iPP/20PA11 is 169.1 °C (T_m(PP)_) and 190.3 °C (T_m(PA11)_); the clear double T_m_ indicated an incompatibility of these two phases, and more details can be seen in Figure S1a for that of iPP/PA11 without a PP-g-MAH additive. Moreover, with the introduction of PA11, T_m(PP)_ was slightly moved to the lower temperature side; a decrease in iPP’s crystallinity resulted, which might be due to the generation of less closely packed iPP crystals. With the addition of PP-g-MAH, the melting behavior of iPP/20PA11/PP-g-MAH was not obviously affected, compared to that of iPP/20PA11.

As for more information on the influence of PA11 on the crystallization behavior of iPP, Figure 2b illustrates an increase in crystallization inlets and cooling peaks in iPP, mainly due to a heterogenous nucleating effect of this second phase. That is, the presence of the PA11 second phase leads to bimodal melting and crystallization of the blend, enhancing the melt strength and foaming performance of iPP, which is similar to the previous reported article [38]. Moreover, the addition of PP-g-MAH could slow this effect, because it adjusted the interconnection of iPP and PA11, so that the cooling temperature peaks of the iPP/20PA11/5PP-g-MAH and iPP/20PA11/10PP-g-MAH shifted towards the low-temperature area, as shown in Figure 2b. Compared to the cooling process of iPP/PA11 with a different PA11 content in Figure S1b, the adding of PP-g-MAH could also affect iPP/PA11’s crystallization property. Because of the functionalization of PP-g-MAH, overcoming the crystallization energy barrier was relatively more difficult than that of iPP/PA11.

**Figure 3:**
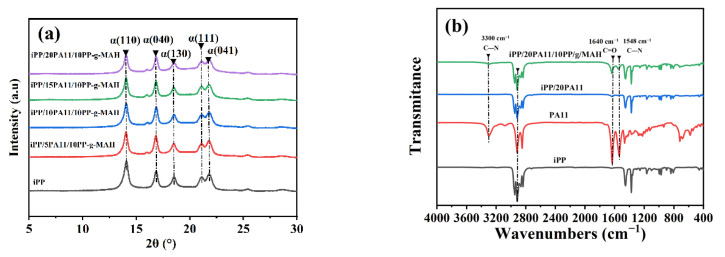
X-Ray graphs (**a**) and FTIR spectrums (**b**) of iPP and iPP/PA11-10PP-g-MAH blends.

Figure 3 shows the X-Ray diffractions and FTIR spectrums for the iPP/PA11/10PP-g-MAH with a different PA11 content. Bragg peaks in Figure 3a at 2θ of 14.1°, 16.9°, 18.6°, 21.1°, and 21.8° refer to planes of (110), (040), (130), (111), and (041) of α crystals, respectively. With the addition of PA11, no new crystals were generated. Similar results in Figure S2a were obtained for iPP and iPP/PA11, with a different PA11 content, but no PP-g-MAH. The addition of PP-g-MAH and PA11 could not change the crystal structure of the iPP/PA11 blends.

The FTIR results in Figure 3b and Figure S2b for iPP, PA11, iPP/20PA11, and iPP/20PA11/10PP-g-MAH reveal that the single addition of PA11 could not generate a new end group. The characteristic peaks at 2918.3 cm^−1^ and 2850.79 cm^−1^ are for symmetric and asymmetric stretching vibration peaks of CH_2_, and a strong 1633.71 cm^−1^ is a typical peak of double-sided stretching vibration of C=O. A 1548.84 cm^−1^ is for a C-N stretching vibration of PA11, and a clear 3300 cm^−1^ peak is for PA11’s NH_2_ vibration. However, because of a 20 wt.% PA11 content and weak surface connection between iPP and PA11, no peak can be seen at 3300 cm^−1^, and only a slight peak at 1633.71 cm^−1^ for iPP/PA11 can be identified. Moreover, with the addition of PP-g-MAH, these three peaks can be obviously seen in iPP/20PA11/10PP-g-MAH, i.e., the maleic anhydride end group of PP-g-MAH could react with the -NH_2_ of PA11, generating an imide group at 1200 to 1300 cm^−1^. In this way, the surface of iPP and PA11 could be strengthened. The distribution of PA11 in the iPP matrix was more uniform, and thus the strength of -NH_2_ was changed by the local molecular vibrating dipole effect.

**Figure 4:**
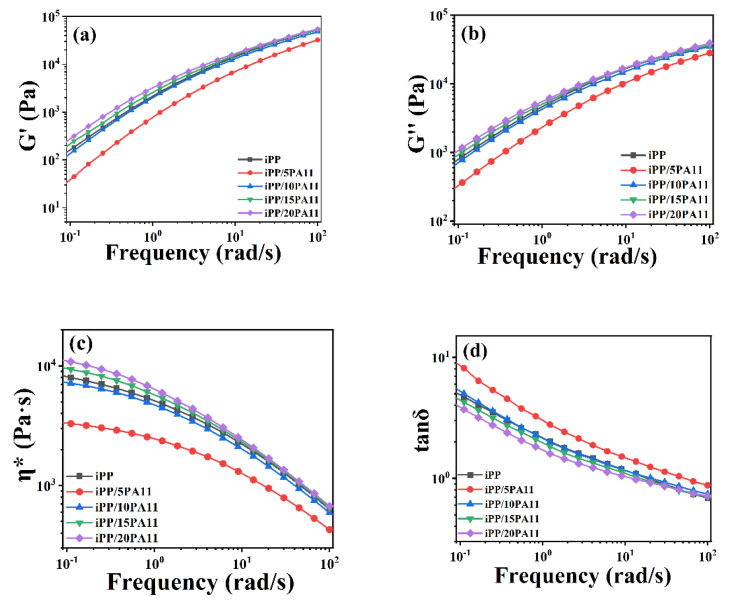
Rheological properties of iPP/PA11 blends at different frequencies (**a**) storage modulus G′ (**b**) viscous modulus G′′ (**c**) complex viscosity η* (**d**) tan δ.

With respect to the rheological behavior of iPP and its blends, as shown in Figure 4, with the presence of PA11, the storage modulus, the loss modulus, and the complex viscosity of the iPP/5PA11 decreased, due to a special lubricating effect of PA11 at a lower PA11 content of 5%. But the effect of more PA11 content of 10, 15, and 20% was different; they all increased by the simple physical crosslinking effect, after overcoming the lubricating influence of a lower PA11 addition. Moreover, with a further addition of PA11, this trend was weakened, as detailed in Figure S3 (because the testing temperature was 200 °C, so the absolute tested values are comparatively small, as shown in Figure S4), through an enhanced bonding between iPP and PA11, which will further affect the mechanical property and foaming behavior of iPP/PA11 blends.

### 3.2. Morphology and Mechanical Property of iPP/PA11/PP-g-MAH Blends

**Figure 5:**
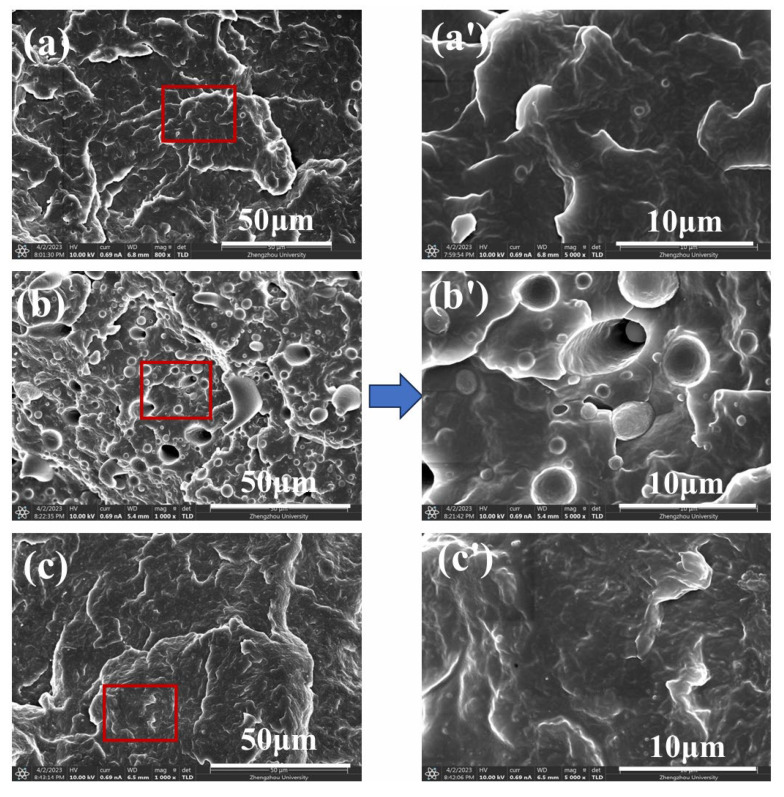
SEM pictures of the fractured iPP/PA11/PP-g-MAH blends with different PA11 content. (**a**) neat iPP, (**b**) iPP/20 PA11, (**c**) iPP/20PA11/10 PP-g-MAH, (**a’**–**c’**) are their magnified pictures within the red boxes.

Figure 5 shows the fractured surfaces of iPP, iPP/20PA11, and iPP/20PA11/10 PP-g-MAH. From Figure 5b,b’ we can see the separated two phases of iPP and PA11, with a poor compatibility (more details in Figure S5). Furthermore, with an introduction of PP-g-MAH, as shown in Figure 5c,c’, the fractured surface reveals a continuous phase state, which can be ascribed to the function of the compatibilizer. This phenomenon is consistent with the former presented FFTIR results, indicating an enhanced compatibility between iPP and PA11.

**Figure 6:**
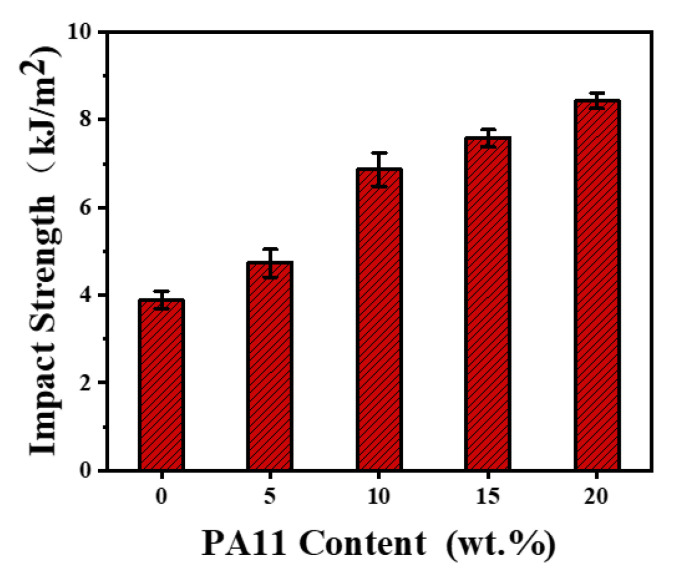
Impact strength of iPP and iPP/PA11/10PP-g-MAH blends with different PA11 content.

**Figure 7:**
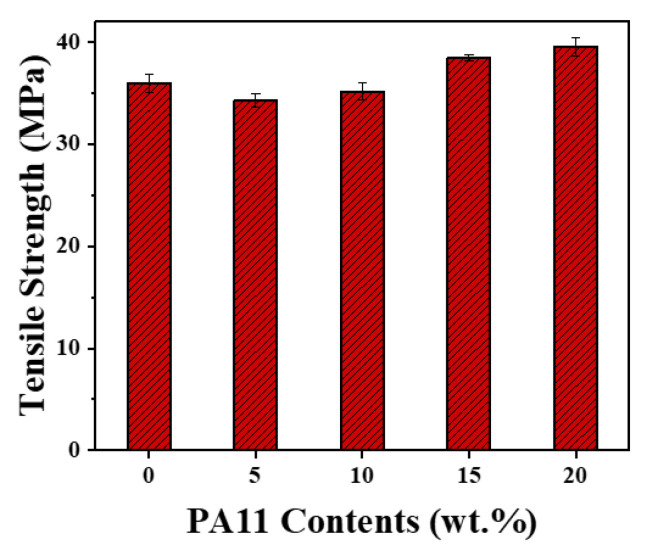
Tensile strength of iPP and iPP/PA11/10PP-g-MAH blends with different PA11 content.

At a given PP-g-MAH content of 10%, the impact strength, tensile strength, and elongation at break of iPP/PA11/10PP-g-MAH increased gradually with the increase in PA11, from 5, 10 to 20 wt.%, as shown in Figure 6 and Figure 7 and Table 2. For example, the values of impact strength, tensile strength, and elongation at break of iPP/20PA11/10PP-g-MAH are 8.43 kJ/m^2^, 39.51 MPa, and 465.87%, compared to that of neat iPP (3.86 kJ/m^2^, 35.98 MPa, 202.41%). An individual 118%, 9.8%, and 130% increment resulted, which could be attributed to the cooperated function of PA11 and PP-g-MAH, as illustrated previously for the structure and morphology development in the iPP/PA11/PP-g-MAH blends. The enhanced molecular bonding and connecting absorbed the energy of the fractured surface during the impact test. These values are more pronounced than those present in Figure S6 and Table S1 for the iPP/PA11 case.

### 3.3. Microcellular Foaming Behavior of iPP/PA11/PP-g-MAH Blends

**Figure 8:**
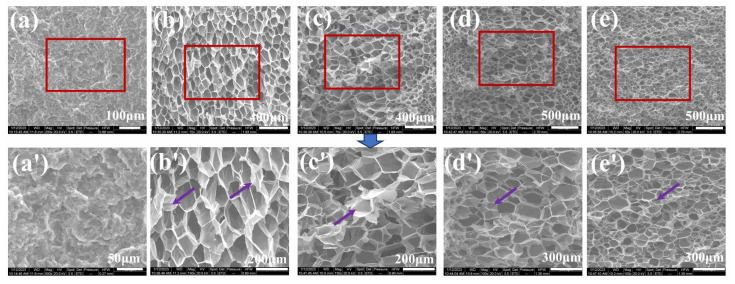
SEM pictures of iPP/PA11/10PP-g-MAH foams with different PA11 content (**a**) neat PP, (**b**) 5PA11, (**c**) 10PA11, (**d**) 15PA11, (**e**) 20PA11, (**a’**–**e’**) are their magnified pictures within the red boxes. The foams are saturated for 30 min at 158 °C and 12 MPa. Arrows in (**b’**–**e’**) point the collapse or rupture of the cells.

**Figure 9:**
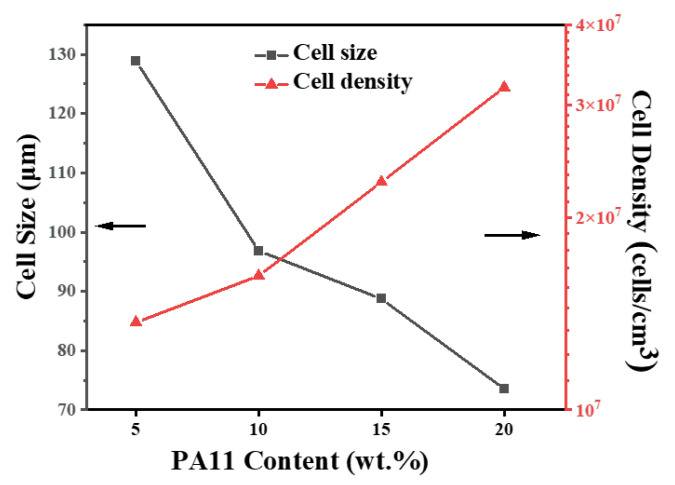
Cell size and cell density of iPP/PA11/10PP-g-MAH foams with different PA11 in Figure 8.

To evaluate the foamability of iPP/PA11/10PP-g-MAH blends, a physical foaming process was conducted using the supercritical CO_2_ as a foaming agent. Firstly, the foaming condition was set at a fixed T_f_ (foaming temperature) of 158 °C, P_f_ (foaming pressure) of 12 MPa, saturating time of 30 min. For neat iPP, under such foaming condition, uniform structure could not be easily obtained; even it could not be easily foamed, due to the higher crystallinity and rigidity of the matrix, as well as the lower CO_2_ solubility. For different PA11 added blends, Figure 8 presents their cell morphologies. With the increase in PA11, under such foaming condition, cooperating with partially melted iPP crystals, the melt strength and cell nucleating ability of the blends improved powerfully, the cell size induced decreased gradually, but the related foam density increased, as calculated and shown in Figure 9. For instance, the average cell size of a foamed iPP/20PA11/10PP-g-MAH is 73.55 μm and 3.19 × 10^7^ cells/cm^3^. Clearly the cell size is smaller than its lower PA11 contented counterparts; however, the cell density is slightly bigger than that of the others. The introduction of PA11 and partially melted PP crystals performed as heterogenous and homogeneous nucleating sites, generating more of the smaller cells if possible, and the increased melt strength was able to help them to withstand the expansion during the degassing process; thus more smaller cells were produced. This is consistent with our former reports; the uniformity of the cells is improved with the increase in PA11, as arrowed in Figure 8 [7,34,35].

**Figure 10:**
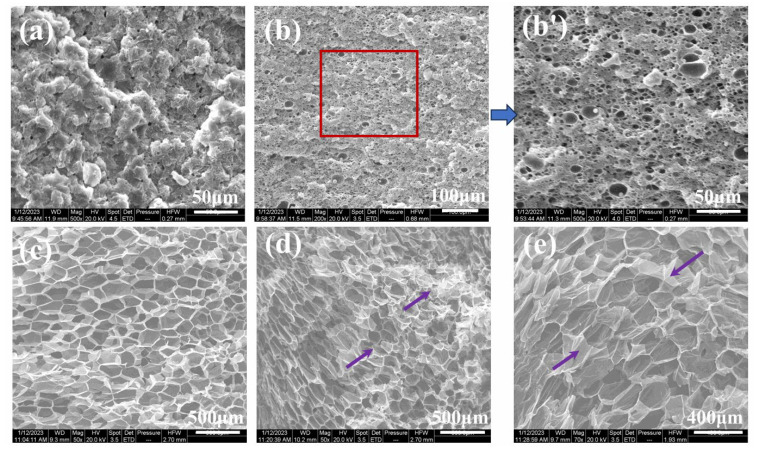
SEM pictures of iPP (**a**) and iPP/5PA11/10PP-g-MAH (**b**–**e**) foams at different foaming temperature of (**a**,**b**) 156 °C, (**c**) 160 °C, (**d**) 162 °C, (**e**) 164 °C, (**b’**) is the magnified picture of (**b**) within the red box. Arrows in (**d**,**e**) point the collapse or rupture development of the cells.

To further investigate the influence of partially melted crystals (adjusted by saturating temperature herein) on the foamability of iPP and iPP/5PA11/10PP-g-MAH blends, different foaming temperatures were set with a fixed pressure of 12 MPa and a given saturation time of 30 min. Morphologies of iPP and iPP/5PA11/PP-g-MAH are shown in Figure 10, and that produced at 158 °C is presented previously in Figure 8b,b’.

**Figure 11:**
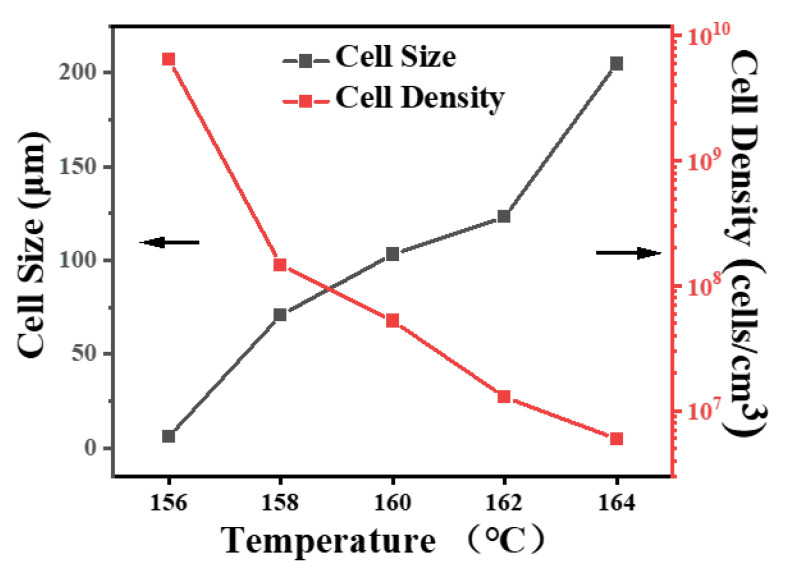
Cell size and cell density of iPP/5PA11/PP-g-MAH foams in Figure 8b and Figure 10.

As an example, under lower foaming temperature of 156 °C, the blend is relatively rigid; less compressed CO_2_ could be absorbed, iPP could not be expanded easily, but smaller-sized iPP/5PA11/10PP-g-MAH cells of 5.4 μm with a cell density up to 6.5 × 10^9^ cells/cm^3^ were achieved (Figure 11), accompanied by an expansion ratio of only 3.08 (Table 3).

At a given pressure of 12 MPa, increasing the saturating temperature to some points means an increased relaxation of the molecules and a decrease in melt strength to some extent, as well as a change in CO_2_ absorption. As a result, with the increase of T_f_, the cell size becomes bigger, while cell density tends to be smaller, and the expansion ratio dramatically increases to 20 more times. At a relatively higher temperature of 164 °C, because of a plasticizing effect of compressed CO_2_, the T_m(PP)_ moved to the lower temperature side, the PP crystals were melted more, and the melt strength greatly decreased. The cell wall was too thin to withstand the degassing of the compressed CO_2_, the uniform cell structure was disrupted, and cell collapse was induced, as arrowed in Figure 10d,e; even its expansion ratio was as high as 37.91. The above results illustrate that with the cooperation of PA11, partially melted iPP crystals were able effectively to influence the cell nucleating, cell growing, and cell structure of the iPP/PA11/PP-g-MAH foams, under certain foaming conditions.

## 4. Conclusions

In summary, as an enhancing strategy to improve the mechanical property and foaming ability of iPP, a second phase of PA11 was used and a PP-g-MAH was added to improve the adhesive strength between iPP and PA11. Because of the reaction compatibility between iPP-g-MAH and PA11, as well as a good connection between iPP and iPP-g-MAH, the separated phase surfaces were fused and enhanced, so the impact strength and elongation at break of iPP/20PA/10iPP-g-MAH obviously increased. Due to the cooperation of these additives and a reasonable foaming condition design, partially melted crystal structure was powerful when applied to adjust the melt strength and foamability of iPP. After being foamed at a suitable condition of T_f_, comparatively uniform iPP/5PA11/10PP-g-MAH cell structures with small cell size and high cell density were obtained as expected. For the case of iPP/PA11/PP-g-MAH, with the increase in PA11 content, at a fixed foaming temperature and pressure, the average cell size of the iPP/PA11/PP-g-MAH foams showed a gradually decreasing trend, and the cell density showed an increasing trend. Moreover, when the PA11 reached a given content of 20 wt.%, the average cell size decreased to 73.6 μm, and the cell density was 3.2 × 10^7^/cm^3^. When the foaming temperature was increased from 156 °C to 164 °C, the average cell size gradually increased from 5.9 μm to 204.8 μm, and the cell density decreased from 6.5 × 10^9^/cm^3^ to 6.0 × 10^6^/cm^3^. Meanwhile, the volume density decreased from 0.290 g/cm^3^ to 0.024 g/cm^3^, but the expansion ratio increased from 3.08 to 37.91. The produced iPP/PA11/PP-g-MAH foams have potential applications in the fields of packaging, household components, and industrial structural parts.

## Figures and Tables

**Table 1:** Melting characteristic parameters of iPP and iPP/20PA11/PP-g-MAH in Figure 2a.

Materials	T_m (PP)_ °C	T_m (PA11)_ °C	ΔH_m (PP)_ (J/g)	ΔH_m (PA11)_ (J/g)	X_c (PP)_ %	X_c (PA11)_ %
iPP	169.2	-	76.46	-	36.6	-
iPP/20PA11	169.1	190.3	56.53	5.23	27.0	2.7
iPP/20PA11/5PP-g-MAH	168.9	189.4	48.30	7.55	23.11	3.9
iPP/20PA11/10PP-g-MAH	167.8	188.7	44.75	11.26	21.41	5.8

**Table 2:** Elongation at break of iPP and iPP/PA11/10PP-g-MAH blends with different PA11 content.

Materials	Elongation at Break/%
iPP	202.41
iPP/5PA11/10 PP-g-MAH	318.28
iPP/10PA11/10 PP-g-MAH	373.78
iPP/15PA11/10 PP-g-MAH	444.77
iPP/20PA11/10 PP-g-MAH	465.87

**Table 3:** Volume foam density and expansion ratio of iPP/5PA11/10PP-g-MAH in Figure 8b and Figure 10.

T_f_ (°C)	Foam Density (g/cm^3^)	Expansion Ratio
156	0.29	3.08
158	0.050	18.73
160	0.042	22.14
162	0.026	34.67
164	0.024	37.91

## Data Availability

The original contributions presented in this study are included in the article/Supplementary Materials. Further inquiries can be directed to the corresponding author.

## Supplementary Materials

The following supporting information can be downloaded at: https://www.mdpi.com/article/10.3390/polym17141952/s1, Figure S1: DSC heating graphs (a) and cooling graphs (b) of iPP and iPP/PA11 blends; Figure S2: X-Ray graphs (a) and FTIR spectrums (b) of iPP and iPP/PA11 blends; Figure S3: Rheological properties (G′ × G″) of iPP/PA11 blends in Figure 4, at different scanning frequencies; Figure S4: Rheological properties of iPP and iPP/PA11/10PP-g-MAH blends at different frequencies (a) storage modulus G′ (b) viscous modulus G″ (c) complex viscosity η* (d) tan δ; Figure S5: SEM pictures of iPP/PA11 with different PA11 content (a) pure iPP, (b) iPP/5PA11, (c) iPP/20PA11, (a’) to (c’) are their magnified pictures; Figure S6: Impact strength (a) and tensile strength (b) of iPP/PA11 blends; Table S1: Elongation at break of iPP/PA11 blends with different PA11 content.
