# Supplementary material for: Mechanical Property and Microcellular Foamability of iPP/PA11/PP-g-MAH Blends
Source: Polymers (Basel). 2025 Jul 16;17(14):1952. doi: 10.3390/polym17141952 (PMC12299155; doi:10.3390/polym17141952)
Supplement: Supplementary file 1 [file polymers-17-01952-s001.zip › polymers-3730802-supplementary.pdf]

Supplementary File

Mechanical property and microcellular foamability of iPP/PA11/PP-g-MAH Blends

Bosi Liu[1,†], Yangzheng Wang[1,2,†], Jingke Pei[3], Qiongdan Fan[1], Kun Li[1], Lele Li[1,*], Xiaoli Zhang[1,*]

[1]School of Materials Science and Engineering, National Engineering Research Center for Advanced Polymer Processing Technology, Zhengzhou University, Zhengzhou, China, 450001

[2]Sinopec (Henan) Refining and Chemical Co., Ltd, Luoyang, China, 471012

[3]Dongfang Electric (Fujian) Innovation Institute Co., Ltd, Fuzhou, China, 350108

†These authors contributed equally to this work

Corresponding Authors: Lele Li (lilele@zzu.edu.cn), Xiaoli Zhang (zhangxl@zzu.edu.cn)

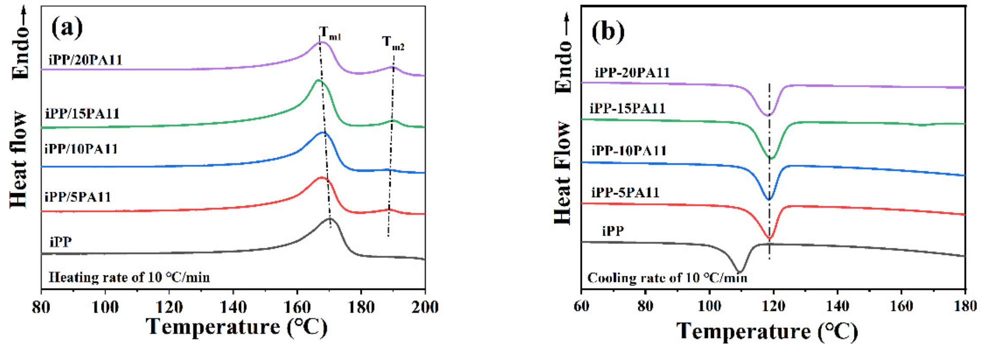

Figure S1. DSC heating graphs (a) and cooling graphs (b) of iPP and iPP/PA11 blends.

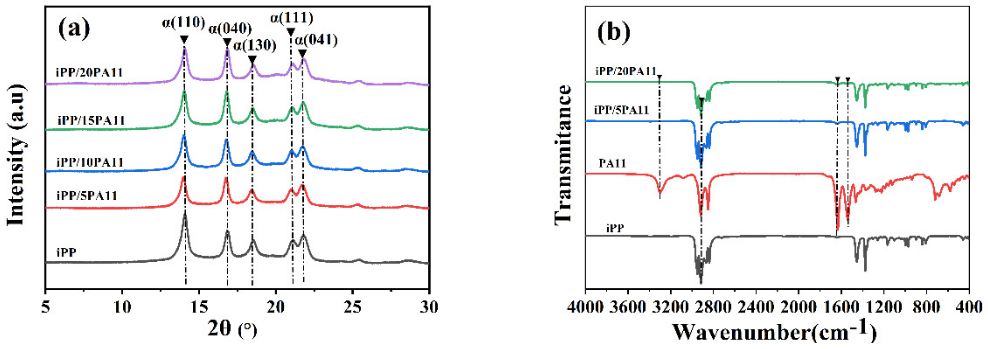

Figure S2. X-Ray graphs (a) and FTIR spectrums (b) of iPP and iPP/PA11 blends.

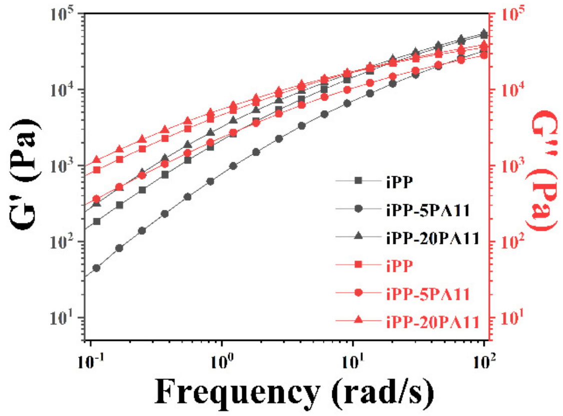

Figure S3. Rheological properties (G′×G″) of iPP/PA11 blends in Figure 4, at different scanning frequencies.

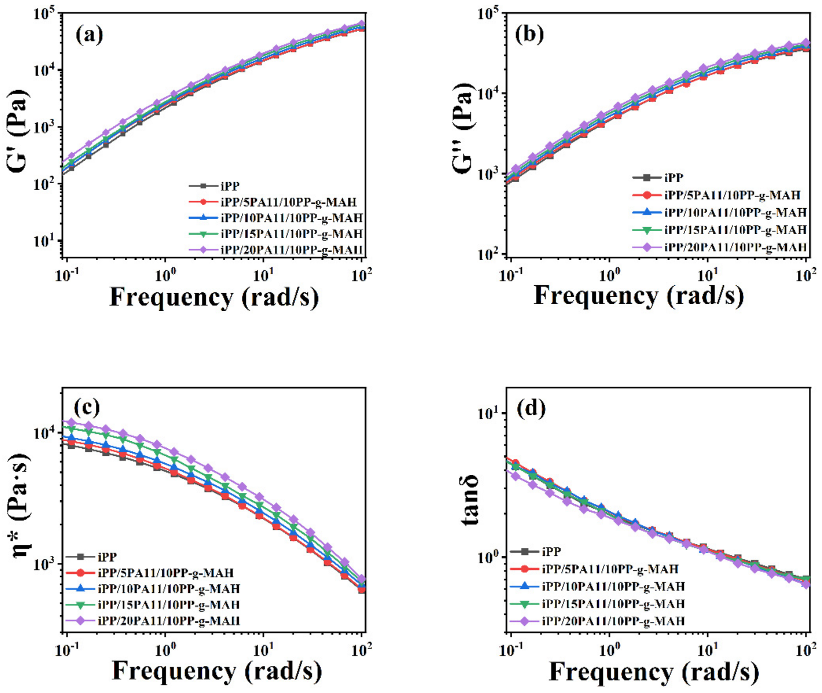

Figure S4. Rheological properties of iPP and iPP/PA11/10PP-g-MAH blends at different frequencies (a) storage modulus G′; (b) viscous modulus G″; (c) complex viscosity η*; (d) tan δ.

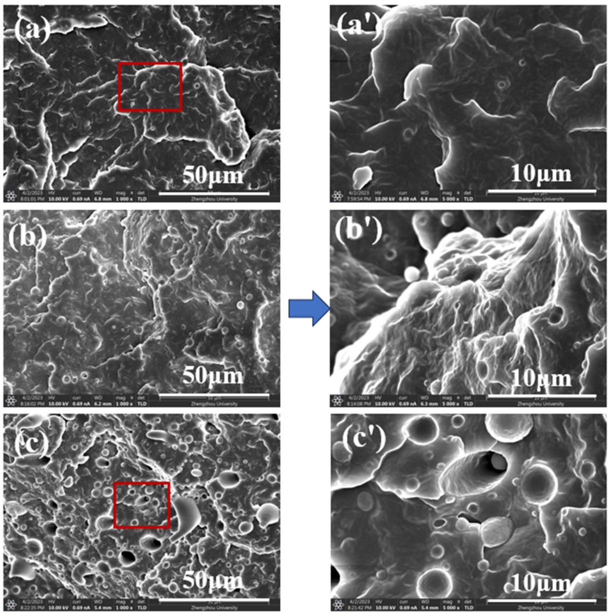

Figure S5. SEM pictures of iPP/PA11 with different PA11 content: (a) pure iPP; (b) iPP/5PA11; (c) iPP/20PA11; (a') to (c') are their magnified pictures within the red boxes.

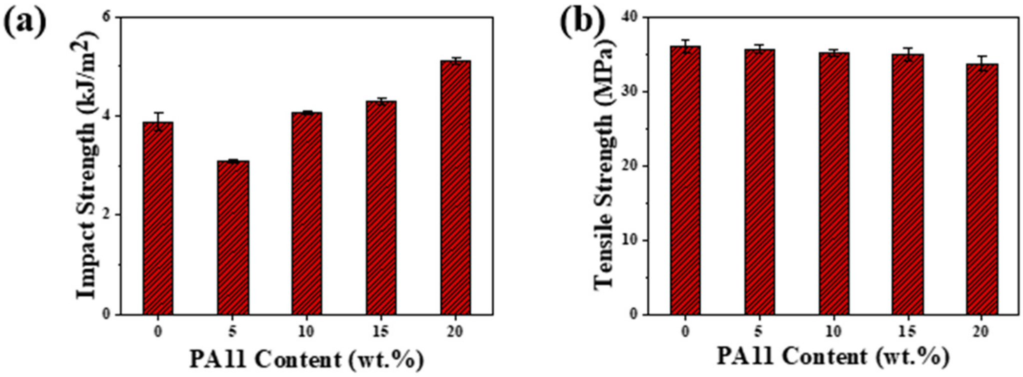

Figure S6. Impact strength (a) and tensile strength (b) of iPP/PA11 blends.

Table S1. Elongation at break of iPP/PA11 blends with different PA11 content.

PA11 content /wt.%	Elongation at break /%
0	202.41
5	150.88
10	154.89
15	116.59
20	69.01
